# Cerebral imaging and neurodevelopmental outcome after entero- and human parechovirus sepsis in young infants

**DOI:** 10.1007/s00431-017-2981-1

**Published:** 2017-09-10

**Authors:** Eveline P. de Jong, Herma C. Holscher, Sylke J. Steggerda, Jeanine M. M. Van Klink, Erika P. M. van Elzakker, Enrico Lopriore, Frans J. Walther, Frank Brus

**Affiliations:** 1 Department of Pediatrics, Leiden University Medical Center, J-6, Albinusdreef 2, 2333 ZA Leiden, The Netherlands; 2 Department of Paediatrics, HAGA hospital, location Juliana Children’s Hospital, Els Borst-Eilersplein 275, 2545 AA The Hague, The Netherlands; 3 Department of Radiology, HAGA hospital, Els Borst-Eilersplein 275, 2545 AA The Hague, The Netherlands; 4 Department of Paediatrics, Division of Neonatology, Leiden University Medical Centre, Albinusdreef 2, 2333 ZA Leiden, The Netherlands; 5 Department of Medical Microbiology, HAGA hospital, Els Borst-Eilersplein 275, 2545 AA The Hague, The Netherlands

**Keywords:** Enterovirus, Human parechovirus, Neurodevelopment, Cerebral imaging

## Abstract

Enterovirus (EV) and human parechovirus (HPeV) are major causes of sepsis-like illness in infants under 90 days of age and have been identified as neurotropic. Studies about acute and long-term neurodevelopment in infants with sepsis-like illness without the need for intensive care are few. This study investigates cerebral imaging and neurodevelopmental outcome following EV and HPeV infection in these infants. We studied infants under 90 days of age who were admitted to a medium care unit with proven EV- or HPeV-induced sepsis-like illness. In addition to standard care, we did a cerebral ultrasound and cerebral magnetic resonance imaging (MRI), as well as neurodevelopmental follow-up at 6 weeks and 6 months and Bayley Scale of Infant and Toddler Development 3rd edition (BSID-III) investigation at 1 year of age. Twenty-six infants, 22 with EV and 4 with HPeV, were analysed. No abnormalities were detected at cerebral imaging. At 1 year of age, two infants had a moderate delay on both the motor and cognitive scale, one on the cognitive scale only and three others on the gross motor scale only.

*Conclusion*: Although our study population, especially the number of HPeV positive infants is small, our study shows that these infants do not seem to develop severe neurodevelopmental delay and neurologic sequelae more often than the normal Dutch population. Follow-up to school age allows for more reliable assessments of developmental outcome and is recommended for further studies to better assess outcome.

**Table:** 

**What is known:** *• Enterovirus and Human Parechovirus infections are a major cause of sepsis-like illness in young infants.* *• After intensive care treatment for EV or HPeV infection, white matter abnormalities and neurodevelopmental delay have been described.*
**What is new:** *• In our ‘medium care’ population, no abnormalities at cerebral imaging after EV- or HPeV-induced sepsis-like illness have been found.* *• At 1 year of age, infants who had EV- or HPeV-induced sepsis-like illness do not seem to develop severe neurodevelopmental delay and neurologic sequelae more often than the normal population.*

## Introduction

Approximately half of all infants younger than 90 days of age that are hospitalized with sepsis-like illness have an infection with enterovirus (EV) and human parechovirus (HPeV) [1–5]. Both EV and HPeV have been identified as neurotropic viruses. Several studies, performed at paediatric or neonatal intensive care units (PICU/NICU), describe cerebral white matter abnormalities in infants with severe EV and HPeV infection. Moreover, long-term impairment, such as neurodevelopmental delay, cerebral palsy and epilepsy, have been reported in survivors after NICU admittance for EV or HPeV infection [6, 7].

However, most infants diagnosed with EV- or HPeV-induced sepsis-like illness do not need intensive care treatment. In this less severely affected population, only two studies, both over 20 years old, about neurodevelopmental follow-up and occurrence of neurologic sequelae exist [8, 9].

The aim of this study was to investigate cerebral imaging and neurodevelopment up to 1 year after infection in infants who had EV- or HPeV-induced sepsis-like illness during their first 90 days after birth.

## Materials and methods

This prospective cohort study was performed at the Juliana Children’s Hospital, The Hague, Netherlands. Patients were included in the study from July 2011 until October 2012, after written informed consent from parents. The study was approved of by the regional medical ethics committee (METC Southwest Holland, ref. 10–158).

We included infants under 90 days of age who were admitted to our medium care unit with proven EV- or HPeV-induced sepsis-like illness. The definition of sepsis-like illness was based on age-specific criteria (Table 1). A positive diagnosis for EV or HPeV infection was made from a positive polymerase chain reaction (PCR) result on either plasma or cerebrospinal fluid (CSF) or both. PCR was performed as reported earlier [5]. Exclusion criteria were congenital anomalies (including cerebral malformations), known or suspected immunologic disorders and previous infection with EV or HPeV.

**Table 1:** Criteria for sepsis-like illness

	0–28 days	29–90 days
Clinical signs and symptoms	One or more: -Toxic appearance -Temperature < 36.0 °C or > 38.0 °C -Feeding problems -Lethargy or irritability -Tachypnea -Tachycardia -Capillary refill > 2 s	One or more: -Toxic appearance -Temperature < 36.0 °C or > 39.0 °C -Fever > 48 h -Lethargy or irritability -Capillary refill > 2 s -Bulging fontanel
Criteria for toxic appearance	Rochester criteria	Yale observation scale > 10

These criteria are a local adaptation of the national guidelines for management of children with fever without source (Dutch association of Paediatrics, NvK)

Baseline patient characteristics at admission were recorded after inclusion. As part of the standard sepsis work-up, all infants underwent blood and (if lumbar puncture was successful) CSF sampling for biochemical analysis, viral analysis for EV and HPeV and bacterial cultures. Herpes simplex virus PCR was performed only on CSF.

### Study protocol

**Fig. 1:**
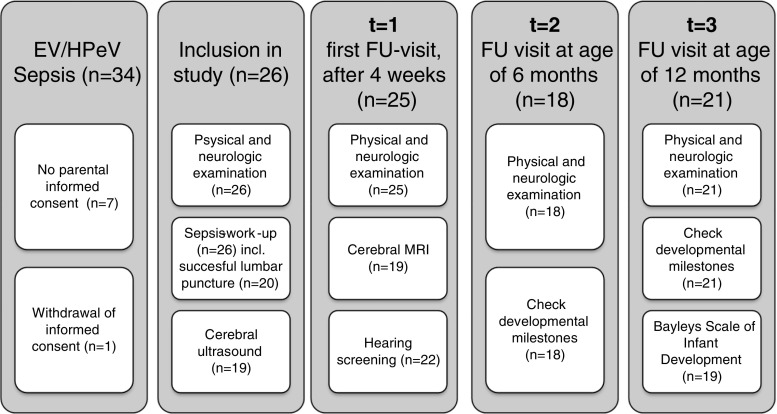
Study protocol and numbers of evaluated patients. Numbers between brackets indicate absolute number of patients

Figure 1 shows the flowchart of the study protocol. One to 2 days after admission, neonatal cranial ultrasound (cUS) views were obtained using a 7.5–10-MHz transducer [10]. Evaluation of cUS was performed using the following criteria: inhomogeneity and/or diffuse echogenicity of the white matter, cystic abnormalities, haemorrhages and echogenicity in the basal ganglia.

Four to 6 weeks after hospital discharge, the infants returned to our outpatient clinic for their first follow-up visit (t1) during which physical and neurological examinations, cerebral magnetic resonance imaging (cMRI) and hearing screening were done. Physical and neurologic examinations were performed by two trained paediatric residents who were unaware of the diagnosis of the patient.

cMRI images were obtained using a Philips 1.0 Tesla scanner (GYROSCAN T10NT PT3000) with 5-mm coupes. Sagittal T1, transversal T1, T2 dual, T2 flair and diffusion-weighted images were obtained. Criteria for abnormalities were as follows: white matter abnormalities (defined as diffuse high signal intensity in the white matter on T2-weighted images and/or punctate white matter lesions), cystic white matter lesions, petechial haemorrhages and signs of white matter atrophy.

During cMRI, no sedation was administered. Infants were fed immediately prior to the MRI, after which most infants fell asleep and were placed on a cushion for minimal movement. All MR-images were reviewed by a paediatric radiologist (HH) and neonatologist (SS), who were unaware of the clinical condition or diagnosis of the patient.

Standard hearing screening was performed during the second or third week of life by means of otoacoustic emission [11], as part of a national newborn screening program. If children were not screened after hospital discharge, we performed automated auditory brain stem response hearing screening during their first follow-up visit.

The second follow-up visit was scheduled at the age of 6 months (*t* = 2) to perform a full physical and neurologic examinations and to complete a checklist for developmental milestones.

At the third follow-up visit at 1 year of age (*t* = 3), the examinations performed at 6 months of age were repeated and the Bayley Scales of Infant and Toddler Development 3rd edition (BSID-III), cognitive and motor scales were investigated, performed by two certified paediatric physiotherapists who were unaware of the diagnosis of the patient. We defined moderate delay as BSID-III cognitive or motor scale scores < − 1 to – 2 SDS, and severe delay as scale scores < − 2 SDS [12, 13].

### Statistics

SPSS was used for data management (PASW statistics version 17.0) and statistical analysis (IBM SPSS statistics version 23.0). Data were checked for normality before analysis, using descriptive statistics and histograms with *z*-scores for skewness and kurtosis. Categorical data are shown as absolute number/total (percentage) and numerical data as median (interquartile range).

*P* values < 0.05 were considered to indicate statistical significance. Mann-Whitney *U* tests and Kruskal Wallis tests were used for numerical data and Fisher’s exact tests for categorical data.

## Results

During the study period, 34 infants met the inclusion criteria, while none fulfilled the exclusion criteria. In eight cases, the parents did not give informed consent for the study. We included 26 infants (22 with EV and 4 with HPeV) in our study. Of the 22 EV positive infants, 10 had a positive PCR in plasma only, in 6/10 cases no or not enough CSF was obtained. EV PCR was positive in CSF only in three cases. In nine, both plasma and CSF were positive. Of the four HPeV infants, two had positive PCR in plasma only due to failure to obtain CSF; the remaining two had positive PCR in both plasma and CSF.

Basic patient characteristics are shown in Table 2. All infants were born at term and had a normal birth weight and normal Apgar scores. None were previously admitted to a hospital ward with perinatal infection. All infants presented to the paediatric emergency room with two or more signs of sepsis-like illness. None of the infants presented with seizures or had abnormal signs at neurologic examination at admission or during their hospital stay.

**Table 2:** Basic patient characteristics

	Total study population (*n* = 26)	EV positive (*n* = 22)	HPeV positive (*n* = 4)
Age at presentation (days)	24 (10–45)	27 (14–49)	10 (8–29)
Duration of symptoms (h)	12 (12–24)	15 (12–24)	12 (9–18)
Feeding problems	15/26 (58%)	14/22 (64%)	1/4 (25%)
Heart frequency (beats/min)	170 (158–180)	169 (155–180)	180 (171–207)
Temperature (°C)	38.6 (38.1–38.9)	38.6 (38.1–39.1)	38.3 (38.0–38.7)
Prolonged capillary refill (> 2 s)	6/26 (23%)	5/22 (23%)	1/4 (25%)
Behavioural symptoms	26/26 (100%)	22/22 (100%)	4/4 (100%)
Lethargy	4/26 (15%)	3/22 (14%)	1/4 (25%)
Irritability	22/26 (85%)	19/22 (86%)	3/4 (75%)
White blood cell count (×10^9^/L)	6.9 (5.5–7.9)	7.5 (5.7–8.6)	5.6 (5.1–5.8)
Thrombocyte count (×10^9^/L)	336 (254–396)	328 (268–395)	356 (197–464)
Haemoglobin (mmol/L)	8.3 (6.5–10.3)	8.2 (6.4–10.2)	8.9 (7.2–10.5)
Glucose (mmol/L)	5.2 (4.9–5.6)	5.2 (4.8–5.6)	5.2 (4.9–5.5)
C-reactive protein (mg/L)	3 (1–6)	3 (1–10)	3 (1–5)

Data are shown as absolute number/total (percentage) or as median (interquartile range). No statistically significant differences were found between EV and HPeV positive infants

The results of the study investigations are shown in Tables 3 and 4. CSF pleocytosis was found in 8/17 (47%) infants with EV infection and in none of the HPeV positive infants. None of the patients had abnormal findings at cUS.

**Table 3:** Neurologic deficit at testing

	Total (*n* = 26)	EV pos (*n* = 22)	HPeV pos (*n* = 4)
Hospital admission (*n* = 26):			
Neurologic examination	0/26	0/22	0/4
CSF pleocytosis^a^	8/20 (40%)	8/17 (47%)	0/3
Cerebral ultrasound	0/20	0/17	0/3
*t* = 1: 4–6 weeks FU visit (*n* = 22):			
Neurologic examination	1/22 (5%)	1/19^b^ (5%)	0/3
Cerebral MRI (cMRI)	0/19	0/16	0/3
Hearing screening	0/22	0/19	0/3
*t* = 2: 6 m FU visit (*n* = 18):			
Neurologic examination (incl. milestones)	1/18 (6%)	1/16 ^c^ (6%)	0/2
*t* = 3: 12 m FU visit (*n* = 20):			
Neurologic examination (incl. milestones)	0/20	0/18	0/2
BSID III^d^:			
Cognitive scale	3/20 (15%) ^e^	2/18 (11%)	1/2 (50%)
Fine motor scale	0/20	0/18	0/2
Gross motor scale	6/20 (30%) ^f^	6/18 (33%)	0/2

Numbers indicate: number of infants with abnormalities / total number of infants tested (percentage)

No statistically significant differences were found between EV and HPeV positive infants

^a^We corrected CSF white blood cell count (WBC) for traumatic puncture if the CSF red blood cell count was >1000 cells/μL, using a 1000:1 ratio [17]. CSF pleocytosis was defined after correction for traumatic puncture as a CSF WBC > 19 cells/μL for children <28 days of age, >9 cells/μL for children 28–58 days of age and >5 cell/μL for children 59–90 days of age [18, 19]

^b^Slight hypertonia of the lower extremities (recovered at subsequent follow-up visits)

^c^Generalized hypotonia with slipping through (recovered at the age of one year)

^d^BSID interpretation: <2 SD = scaled score < 4 (severe delay), −1 to −2 SD = scaled score 4–7 (moderate delay)

^e^Two infants with moderate cognitive delay also had moderate gross motor delay (Table 4)

^f^5/6 infants had a scaled score of 4–7, one had <4

**Table 4:** Clinical details and findings on cerebral imaging in infants with abnormalities at neurologic evaluation

	Sex, age (days)	Symptoms	Diagnosis Plasma PCR CSF PCR Pleocytosis	cUS cMRI	Neur. exams 1. 4–6 weeks after infection 2. Age 6 months 3. Age 12 months	BSID-III^a^; overall result • Cognitive—scaled score • Fine motor—scaled score • Gross motor—scaled score
Case #1	Male 25	Fever, irritability, feeding problems	EV Positive Failed Failed	Normal Normal	1. Slight hypertonia of lower extremities 2. Normal 3. Normal (milestones: slight lead)	Normal • Cognitive—11 • Fine motor—8 • Gross motor—16
Case #2	Female 42	Fever, irritability	EV Positive Failed Failed	Failed Normal	1. Normal 2. Generalized hypotonia. Normal milestones 3. Full recovery, no hypotonia. Normal	Moderate delay • Cognitive—6 • Fine motor—9 • Gross motor—5
Case #3	Female 10	Fever, irritability, feeding problems	EV Negative Positive Yes	Normal Normal	1. Normal 2. Normal 3. Normal	Moderate delay • Cognitive—6 • Fine motor—8 • Gross motor—6
Case #4	Female 6	Fever, lethargy	HPeV Positive Failed Failed	Failed Normal	1. Normal 2. Normal 3. Normal	Moderate delay • Cognitive—6 • Fine motor—10 • Gross motor—7
Case #5	Male 22	Fever, irritability, feeding problems	EV Positive Negative Yes	Normal Normal	1. Normal 2. Normal 3. Normal (milestones: slight delay gross motor development)	Moderate delay • Cognitive—7 • Fine motor—9 • Gross motor—4
Case #6	Male 15	Fever, irritability	EV	Normal	1. Normal	Moderate delay
			Positive	Normal	2. Normal	• Cognitive—10
			Positive		3. Normal	• Fine motor—11
			No			• Gross motor—5
Case #7	Male 50	Fever, irritability	EV	Normal	1. Failed	Moderate delay
			Positive	Failed	2. Normal	• Cognitive—9
			Positive		3. Normal	• Fine motor—9
			Yes			• Gross motor—6
Case #8	Male 69	Fever, irritability, feeding problems	EV	Failed	1. Normal	Severe delay
			Positive	Normal	2. Normal	• Cognitive—10
			Positive		3. Normal	• Fine motor—14
			No			• Gross motor—3

^a^< 2 SD = scaled score < 4, − 1 to − 2 SD = scaled score 4–6, − 1 to + 1 SD = scaled score 7–13, + 1 to + 2 SD = scaled score 13–15, > + 2 SD = scaled score > 15

At the first follow-up visit 4–6 weeks after infection, 22/26 (85%) infants were evaluated. All had normal findings at physical examination and hearing tests. Neurologic examination was abnormal in one case (1/22, 5%) showing slight hypertonia of the lower extremities (case #1, Table 4). In this infant, hypertonia had recovered completely at subsequent follow-up visits. Cerebral MRI was successfully performed in 19/26 (73%) of infants and showed no abnormal findings.

At 6 months of age, 18/26 (69%) infants were re-examined. One infant (case #2, Table 4) had general hypotonia and slipping through, but milestones were normal. In this infant, hypotonia had recovered at 12 months of age, but on both the BSID-III cognitive and motor scales, this infant had moderate developmental delay.

At 12 months of age, 20/26 (77%) infants were assessed with the BSID-III. Two of them had experienced an HPeV infection and 18 had an EV infection. The remaining six infants were lost to follow-up, two because of moving of the family and four for unknown reason. One infant had a severe delay on the gross motor scale (case #8, Table 4), the other tested domains were normal. Two had a moderate delay on both the motor and cognitive scale (cases #2–3, Table 4), one on the cognitive scale only (case #4, Table 4) and the others on the gross motor scale only (cases #5–7, Table 4). None of these infants had abnormalities on cerebral imaging. All infants that had a delay at BSID-III testing were thereafter treated with physiotherapy.

## Discussion

This study reports on cerebral imaging and neurodevelopmental outcome of young infants with EV- or HPeV-induced sepsis-like illness in their first 90 days of life, who did not need paediatric or neonatal intensive care admission. We investigated the presence of neurologic signs and symptoms at three time points after EV- or HPeV-induced sepsis-like illness during the first year of life. In this relatively small study, one infant had a severe gross motor neurodevelopmental delay at 1 year of age. Two infants had a moderate delay on both the gross motor and cognitive scales, one on the cognitive scale only and three others on the gross motor scale only. Two infants had transient mild abnormalities at neurologic examination.

Neurodevelopment after EV infection has been reported previously, but only two studies investigated this after EV sepsis/meningitis in the first 90 days of life. Baker et al. describe subtle deficits in receptive language processing, but no differences in motor or cognitive development in their study group of 16 infants compared to healthy matched controls during a 3-year follow-up period [8]. In 1981, a case control study including nine children after EV meningitis also reported deficits in receptive language functioning compared to nine healthy matched controls. No differences in head circumference, sensorineural hearing loss or intellectual functioning between groups were found [9]. Our population was too young to investigate language development reliably. However, none of the children required speech-language therapy. Comparing our results with these studies is difficult, since different (versions of) developmental tests were used 20 years ago.

Only a few studies reported on the neurodevelopmental outcome following HPeV infection. One Australian study describes of nine young infants with a median age at diagnosis of 13 days requiring intensive care treatment for HPeV encephalitis and reports ‘significant or some developmental concern’ in 7/9 (78%) of them 1 year after infection, two were diagnosed with cerebral palsy and one with visual impairment. All had scores below the cut-off of < 2 SD below the population mean in the gross motor subscale of the Ages and Stages questionnaire [14]. This study shows more severe sequelae than our study does, which can be expected from this more ill population.

Our study was performed before a Dutch normation for the BSID-III was available; therefore, we used the USA normation. One recent study showed that the USA normation overestimates cognitive and fine motor development in Dutch healthy infants, but significantly underestimates the gross motor development. This leads to a much higher percentage of infants with low gross motor scores; 43% of Dutch infants scored < 1 SD and 15% < 2 SD [12]. Therefore, in our cohort, no major difference in neurodevelopmental delay compared to healthy Dutch children of the same age was detected [12].

We found no abnormalities on cUS during admission and on cMRI 4 to 6 weeks after the infection. A retrospective Norwegian study described neurodevelopmental outcome and cerebral imaging in 15 HPeV positive infants. They were admitted to a level 2 or 3 hospital ward. In three cases, a cMRI was obtained showing signs of white matter necrosis in two of them. One infant recovered completely within 8 days; the other had normal neurodevelopment at 1 year of age [15].

Two studies describe cerebral imaging in detail in NICU-admitted infants with HPeV infection. One study reports white matter damage and severe periventricular echogenicity in 9 out of 10 infants, resulting to severe neurodevelopmental delay in 2 infants and minor deficits in 2 others. Two of these infants were also born extremely premature and although cUS abnormalities developed after infection with HPeV, these might not be the only cause of cerebral damage [7]. Another study describes normal sequential cUS imaging in 11 HPeV positive infants admitted to a NICU [16].

Cerebral imaging data of EV positive infants is only available from (neonatal) intensive care studies. In 2006, Verboon et al. reported six infants, five infants had periventricular echogenicity on cUS and cMRI showed diffuse high signal intensity in the white matter, punctate white matter lesions or cystic leucomalacia in all of them. Two infants developed cerebral palsy and epilepsy, one was suspect for neurodevelopmental delay at 18 months of age and three developed normally [6].

A major difference with previous studies is that we did not investigate a NICU population and none of the infants in our study developed seizures. Our patients were somewhat older (up to 90 days of age at admission) and less ill than the NICU population. Possibly, the younger and more severely ill infants with haemodynamic instability and/or prolonged seizures, needing intensive care treatment, are at higher risk for development of neurologic sequelae.

Our study has its limitations, including the relatively small cohort and missing values. Although previous studies included cohorts of similar size, the number of patients is too still small to allow firm conclusions, especially in terms of HPeV infection (*n* = 4, at 1 year of age *n* = 2). We followed our study population only for 1 year and therefore it is possible that some neurodevelopmental problems may appear later in life or will spontaneously resolve as children develop. Our data must therefore be interpreted with caution and longer follow-up studies are necessary. Follow-up to school age allows for more reliable assessments of developmental outcome.

We did not perform typing of EV and HPeV, as this has no consequences for treatment. Nevertheless, it would be interesting to define if the more pathogenic EV or HPeV serotypes [1, 3, 4] were present in our population. This might affect treatment if IVIG is used and might allow for more targeted follow-up of these specific infants.

Finally, MRI scanning was not performed during the acute stage of the illness but 4–6 weeks after the infection. Therefore, we may have missed possible abnormalities on the diffusion-weighted images that disappear after the acute phase and were only able to look for subacute signs of white matter injury such as cystic white matter lesions, focal/punctate white matter lesions, delayed myelination, dilatation of the lateral ventricles and other signs of white matter volume loss and did not find any of these white matter abnormalities in our population. Of note, the MRI sequences used in this study had a scan thickness of 5 mm and therefore we may have missed some of the more subtle lesions.

Young infants with sepsis-like illness are regularly admitted to paediatric wards, especially during the EV and HPeV epidemic season in late spring and summer. We show data from cerebral imaging in a large proportion of our study population (19/26). Further, negative hearing screening tests and results from our systematic physical and neurologic examinations are valuable information for paediatricians.

Our study shows that these infants do not seem to develop neurodevelopmental delay and neurologic sequelae more often than the normal population after a 1-year follow-up period. But, considering our studies’ limitations, larger and longer follow-up studies are needed to provide a more definite advice.

## Abbreviations

BSID-III: Bayley Scales of Infant and Toddler Development 3rd edition
cMRI: Cerebral magnetic resonance imaging
CSF: Cerebrospinal fluid
cUS: Cranial ultrasound
EV: Enterovirus
HPeV: Human parechovirus
NICU: Neonatal intensive care unit
PICU: Paediatric intensive care unit
PCR: Polymerase chain reaction
WBC: White blood cell count,
USA: United States of America

## References

[1] Rotbart HA, McCracken GH, Whitley RJ, Modlin JF, Cascino M, Shah S, Blum D (1999). Clinical significance of enteroviruses in serious summer febrile illnesses of children. Pediatr Infect Dis J.

[2] Verboon-Maciolek MA, Krediet TG, Gerards LJ, Fleer A, van Loon TM (2005). Clinical and epidemiologic characteristics of viral infections in a neonatal intensive care unit during a 12-year period. Pediatr Infect Dis J.

[3] Boivin G, Abed Y, Boucher FD (2005). Human parechovirus 3 and neonatal infections. Emerg Infect Dis.

[4] Benschop KS, Schinkel J, Minnaar RP, Pajkrt D, Spanjerberg L, Kraakman HC, Berkhout B, Zaaijer HL, Beld MG, Wolthers KC (2006). Human parechovirus infections in Dutch children and the association between serotype and disease severity. Clin Infect Dis.

[5] De Jong EP, Van den Beuken MGA, Elzakker EPM, Wolthers KC, Sprij AJ, Lopriore E, Walther FJ, Brus F (2017) Epidemiology of sepsis-like illness in young infants: major role of enterovirus and human parechovirus. Pediatr Infect Dis J. doi:10.1097/INF.0000000000001718 10.1097/INF.0000000000001718 28763426

[6] Verboon-Maciolek MA (2006). White matter damage in neonatal enterovirus meningoencephalitis. Neurology.

[7] Verboon-Maciolek MA, Groenendaal F, Hahn CD, Hellmann J, van Loon AM, Boivin G, de Vries LS (2008). Human parechovirus causes encephalitis with white matter injury in neonates. Ann Neurol.

[8] Baker RC, Kummer AW, Schultz JR, Ho M, Gonzalez del Rey J (1996). Neurodevelopmental outcome of infants with viral meningitis in the first three months of life. Clin Pediatr (Phila).

[9] Wilfert CM, Thompson RJ, Sunder TR, O'Quinn A, Zeller J, Blacharsh J (1981). Longitudinal assessment of children with enteroviral meningitis during the first three months of life. Pediatrics.

[10] Gv W-M (2007). Neonatal cranial ultrasonography: guidelines for the procedure and atlas of normal ultrasound anatomy.

[11] van der Ploeg CPB, Uilenburg NN, Kauffman-de Boer MA, Oudesluys-Murphy AM, Verkerk PH (2012). Newborn hearing screening in youth health care in the Netherlands: national results of implementation and follow-up. Int J Audiol.

[12] Steenis LJ, Verhoeven M, Hessen DJ, van Baar AL (2015). Performance of Dutch children on the Bayley III: a comparison study of US and Dutch norms. PLoS One.

[13] Lees CC, Marlow N, van Wassenaer-Leemhuis A, Arabin B, Bilardo CM, Brezinka C, Calvert S, Derks JB, Diemert A, Duvekot JJ, Ferrazzi E, Frusca T, Ganzevoort W, Hecher K, Martinelli P, Ostermayer E, Papageorghiou AT, Schlembach D, KTM S, Thilaganathan B, Todros T, Valcamonico A, GHA V, Wolf H 2 year neurodevelopmental and intermediate perinatal outcomes in infants with very preterm fetal growth restriction (TRUFFLE): a randomised trial. The Lancet 385(9983):2162–2172. doi:10.1016/S0140-6736(14)62049-3 10.1016/S0140-6736(14)62049-3 25747582

[14] Britton PN, Dale RC, Nissen MD, Crawford N, Elliott E, Macartney K, Khandaker G, Booy R, Jones CA (2016). Parechovirus encephalitis and neurodevelopmental outcomes. Pediatrics.

[15] Skram MK, Skanke LH, Krokstad S, Nordbo SA, Nietsch L, Dollner H (2014). Severe parechovirus infection in Norwegian infants. Pediatr Infect Dis J.

[16] Davis J, Fairley D, Christie S, Coyle P, Tubman R, Shields MD (2015). Human parechovirus infection in neonatal intensive care. Pediatr Infect Dis J.

[17] Greenberg RG, Smith PB, Cotten CM, Moody MA, Clark RH, Benjamin DK (2008). Traumatic lumbar punctures in neonates: test performance of the cerebrospinal fluid white blood cell count. Pediatr Infect Dis J.

[18] Kliegman RM SB, St. Geme J, Schor NF (2016) Nelson textbook of pediatrics, vol 2, 20th edn. Elsevier Inc, Philadelphia, p 2799

[19] Kestenbaum LA, Ebberson J, Zorc JJ, Hodinka RL, Shah SS (2010). Defining cerebrospinal fluid white blood cell count reference values in neonates and young infants. Pediatrics.

